# Supplementary material for: Good long-term outcomes of primary sclerosing cholangitis in childhood
Source: JHEP Rep. 2024 May 25;6(8):101123. doi: 10.1016/j.jhepr.2024.101123 (PMC11321284; doi:10.1016/j.jhepr.2024.101123)
Supplement: Multimedia component 1 [file mmc1.pdf]

Good long-term outcomes of primary sclerosing cholangitis in childhood

Anna Jerregård Skarby, Thomas Casswall, Annika Bergquist, Lina Lindström

Table of contents

Table S1……………………………………………………………………………...….2

Table S1. Cox PH regression with c-statistics to evaluate performance of SCOPE risk index at 5 years from diagnosis of PSC and at last date of follow-up.

	5 years after diagnosis				Last follow-up			
	**HR**	**95% CI**	***p*-value**	**c-statistics**	**HR**	**95% CI**	***p*-value**	**c-statistics**
**Any hepatobiliary complications**	3.131	1.38-7.33	**0.009**	0.751	1.380	0.88 - 2.19	0.171	0.590
**Liver transplantation/ Death from liver disease**	2.873	0.61-13.59	0.183	0.732	2.350	1.18 - 4.66	**0.015**	0.701

SCOPE index = Sclerosing Cholangitis Outcomes in Pediatrics index, Any hepatobiliary complications = any of portal hypertension, biliary complications, hepatobiliary cancer, liver transplantation or death of liver disease, HR = Hazard ratio, CI = confidence interval, c-statistic = concordant statistic
p value <0.05 was considered significant. Significant *p* values are in bold.
