# Supplementary material for: Good long-term outcomes of primary sclerosing cholangitis in childhood
Source: JHEP Rep. 2024 May 25;6(8):101123. doi: 10.1016/j.jhepr.2024.101123 (PMC11321284; doi:10.1016/j.jhepr.2024.101123)
Supplement: Multimedia component 4 [file mmc4.pdf]

Good long-term outcomes of primary sclerosing cholangitis in childhood

Anna Jerregård Skarby[1,*], Thomas Casswall[2], Annika Bergquist[3], Lina Lindström[4]

JHEP Reports **2024**. vol. 6 | 1–9

Check for updates

Background & Aims: Primary sclerosing cholangitis (PSC) is a rare progressive liver disease associated with inflammatory bowel disease (IBD). It is usually diagnosed in adults but can also present in children. Data on long-term outcomes of pediatric PSC are limited. Our aim was to study the natural history of pediatric PSC in Sweden.

Methods: This is a cohort study, including all children (<18 years), diagnosed with PSC between January 2000 and December 2015 at the Pediatric Liver Unit at Karolinska University Hospital, Stockholm. Patients were followed until liver transplantation, death or last date of follow-up (August 2021).

Results: We identified 124 children with a median age of 14 (1.9–17.8) years at PSC diagnosis. Sixty percent were boys, 93% had IBD. Median follow-up time was 13 years (5.7–21.6). Overall event-free survival in the cohort was 91% (95% CI 0.84–0.95) at 5 years and 77% (95% CI 0.68–0.84) at 10 years after diagnosis. Autoimmune hepatitis (AIH) was present in 31% (n = 39). Portal hypertension developed in 13% (n = 16), biliary complications in 24% (n = 30), cholangiocarcinoma (CCA) in 0.8% (n = 1), while 13% (n = 16) underwent liver transplantation and three patients died. Transplant-free survival was 91% after 10 years. Individuals with a high SCOPE index at diagnosis had a 2.3-fold increased risk of requiring liver transplantation (hazard ratio 2.35, 95% CI 1.18–4.66, c-statistics = 0.70). Patients with an additional diagnosis of autoimmune hepatitis had slightly higher risk of reaching transplantation during follow-up (hazard ratio 2.85, 95% CI 1.06–7.67, *p* = 0.038).

Conclusions: Children diagnosed with PSC have a good prognosis during the first decade after diagnosis. A high SCOPE index at diagnosis was associated with a less favorable outcome.

© 2024 The Authors. Published by Elsevier B.V. on behalf of European Association for the Study of the Liver (EASL). This is an open access article under the CC BY license (http://creativecommons.org/licenses/by/4.0/).

Introduction

Primary sclerosing cholangitis (PSC) is a rare progressive chronic liver disease, characterized by biliary inflammation leading to bile duct strictures, cholestasis, periductal fibrosis and cirrhosis.[1] PSC is usually diagnosed in adults but can more rarely present in children. Patients with PSC have a high lifetime risk of developing recurrent cholangitis, end-stage liver disease, and hepatopancreatobiliary malignancies, particularly cholangiocarcinoma (CCA).[1] There is currently no proven medical treatment that can halt disease progression. The natural history of adult PSC is well described, and the transplant-free survival is reported to be up to 21 years in population-based cohorts.[2] In children, the disease is even more rare, and the natural history of childhood-onset PSC is less well described than in adults. One study including 781 children with PSC reports progressive liver disease in 53% of the children 10 years after diagnosis.[3] The incidence rate of pediatric PSC has been estimated to be 0.2 per 100,000 per year.[4]

PSC is closely associated with inflammatory bowel disease.[5] The prevalence of IBD in children with PSC has been reported to be about 80%, similar to that in adults.[3,6] Presence of AIH-like features are also common, especially in childhood-onset PSC. The prevalence of AIH features in children is reported to be 25–72% compared to 7–14% in adults.[7,8]

Data on long-term outcomes of patients with PSC diagnosed in childhood is scarce.[4] Early pediatric studies are mostly single center with limited follow-up time.[3,9–11] The first and largest study on the natural history of pediatric PSC was published in 2017. This study collected data from 781 children with PSC from 36 centers in North America, Europe (Sweden not represented), the Middle East and Asia, showing an occurrence of liver transplantation of 14%; the median follow-up time was 4.4 years.[3]

Based on this cohort data, the first pediatric PSC prognostic tool, Sclerosing Cholangitis Outcomes in Pediatrics (SCOPE) index, was developed in 2020.[12] This tool includes total bilirubin, albumin, platelet count, gamma-glutamyltransferase (GGT) and cholangiography and aims to predict liver transplantation, death and/or any hepatobiliary complications within 1 to 5 years of follow-up.[12]

In this single-center cohort of patients with PSC diagnosed during childhood, we aim to describe the presentation of PSC in children, the natural history of the disease and the long-term prognosis.

* Corresponding author. Address: Karolinska Institutet, 141 86 Stockholm, Sweden. Tel.: +46 790 630 256.
E-mail address: anna.jerregard.skarby@ki.se (A. Jerregård Skarby).
https://doi.org/10.1016/j.jhepr.2024.101123

Materials and methods

Study population

This is a cohort study including all consecutive children (age<18 years) diagnosed with PSC with or without concomitant AIH at the Pediatric Gastroenterology, Hepatology and Nutrition Unit, a tertiary referral clinic, at Karolinska University Hospital, Stockholm, Sweden. The patients included covered approximately 80% of the regions in Sweden. Fifty-seven percent of the patients were from Stockholm. One hundred and forty-four patients were identified from the local visit register using the ICD-10 codes K830A (sclerosing cholangitis), K830X (cholangitis unspecified) and K830 (cholangitis, including bacterial cholangitis due to causes other than PSC). All children with a confirmed diagnosis between 01-01-2000 and 12-31-2015 were included, Fig 1, and followed from diagnosis until last date of follow-up (08-31-2021) or death.

The cohort was divided into two major subgroups: 1) patients with PSC alone and 2) patients with PSC and an additional diagnosis of AIH. Analyses were made on the total cohort, and on the subgroups separately.

Data collection

Clinical and radiological data was collected at diagnosis and during follow-up by retrospective chart review. Histology and biochemistry were collected at diagnosis and last follow-up. Liver biopsy reports made by experienced pathologists in pediatric hepatology were reviewed, but no re-evaluation was made. The following variables were registered: age at diagnosis, sex, biochemistry, PSC phenotype, liver histology (stage of fibrosis, grade of inflammation, presence of interphase hepatitis, histology concordant with PSC), cholangiography (magnetic resonance cholangiopancreatography [MRCP] or endoscopic retrograde cholangiopancreatography [ERCP]), transient elastography, radiology (cirrhosis, deformed liver, splenomegaly, esophageal varices, ascites, dominant strictures), presence of AIH, presence and date of IBD diagnosis, IBD phenotype, colectomy, treatment at time of last follow-up, alcohol intake at last follow-up, extrahepatic autoimmune comorbidity, first date of portal hypertension, biliary complications, cancer, liver transplantation, date and cause of death.

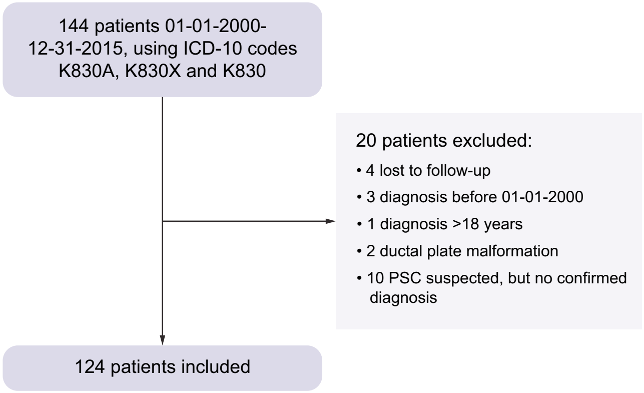

Fig. 1. Flow chart over the inclusion process. K830A, sclerosing cholangitis; K830X, cholangitis unspecified; K830, cholangitis, including bacterial cholangitis due to causes other than PSC. PSC, primary sclerosing cholangitis.

Establishing diagnosis

We used the EASL guidelines to confirm a diagnosis of PSC.[13] If MRCP/ERCP was normal but concomitant presence of histological features consistent with PSC were found, small duct PSC was confirmed. Liver biopsies were performed in all but one patient as a part of the diagnostic process. The date of MRCP/ERCP or liver biopsy was considered to be the date of diagnosis. If PSC was suspected, but MRCP inconclusive, the first MRCP was considered the date of diagnosis provided that the second MRCP or a liver biopsy showed pathological findings typical for PSC. Diagnosis of AIH was assessed using the simplified diagnostic criteria for AIH validated for children, classifying patients into probable or definite AIH.[14] In cases where biochemical and immunological data was incomplete, the presence of a clinical diagnosis of AIH was accepted.

Outcomes

The following outcomes were considered:

1) Complications related to portal hypertension, defined as at least one of the following: ascites in need of medical treatment or intervention, hepatic encephalopathy, esophageal varices or splenomegaly and at the same time low platelet count. Cut-off for low platelet count was platelets under the lowest age-specific reference values.
2) Biliary complications defined as clinical cholangitis with biliary strictures requiring stenting, balloon dilatation or drainage.
3) Cancer; CCA and hepatocellular carcinoma.
4) Liver transplantation.
5) Death from liver disease.

All children were followed from diagnosis to each of the five endpoints or to the last date of follow-up.

SCOPE risk index

SCOPE risk index was calculated at diagnosis, using the variables total bilirubin, albumin, platelet count, GGT and findings on cholangiography (normal or large duct involvement).[12] The patients were divided into risk groups based on SCOPE index points: low (0–3), medium (4–5) or high risk (6–11) for adverse liver events within 5 years from diagnosis.

Statistical analysis

Demographical and clinical characteristics at diagnosis are presented as absolute numbers, median and range. Clinical characteristics and long-term outcomes were presented on the cohort as a whole and divided into the PSC alone and PSC-AIH groups, and comparisons between these were made with Fisher's exact test. Survival analyses of the different outcomes and a composite outcome of time free from any of the five endpoints during follow-up were performed on the total cohort with the Kaplan-Meier method. Patients presenting with portal hypertension at the time of diagnosis were excluded from survival analysis for the endpoint portal hypertension and the composite endpoint. Survival analyses comparing patients with PSC alone to those with PSC-AIH were performed using the Kaplan-Meier method and log-rank test. Influence on outcome depending on sex, age at PSC diagnosis (<12 or ≥12 years), GGT at diagnosis (>36.0 U/L or ≤36.0 U/L), PSC phenotype, concomitant AIH and IBD was evaluated with

univariate Cox proportional-hazards regression analysis. The variables in the univariate analysis were also included in a multivariable model for further evaluation of factors associated with a more rapid disease progression. Pairwise *post hoc* testing to compare all possible pairs of the different IBD-phenotypes and the patients without a diagnosis of IBD was made using the Tukey method. Validation of the SCOPE risk index at 5 years after diagnosis and at the last date of follow-up was performed using Cox proportional-hazards regression analysis using c-statistics. *P* values <0.05 were considered statistically significant. Statistical analyses were performed with STATA/BE 17.0 and R statistical programming software version 4.2.3 and RStudio.

Ethical approval

The study was approved by the Swedish Ethical Review Authority (Dnr: 2021-06631-01).

Results

Clinical characteristics at diagnosis

Out of 144 identified possible participants, we identified 124 patients with a confirmed diagnosis of PSC with or without an additional diagnosis of AIH. Median age at diagnosis was 14 years (1.9–17.8). Median individual time of follow-up was 13 years (5.7–21.6). Median age at last follow-up was 26.8 years (14.0–37.2) and the total number of person-years was 1,631.

Sixty percent (n = 75) of participants were boys, and concomitant AIH was present in 31% (n = 39), all AIH type 1. There was a male predominance (66%, n = 56) in the PSC subgroup. In the PSC and AIH-subgroup the gender distribution was equal (male 49% [n = 19] and female 51% [n = 20]). Large duct PSC was present in 93% (n = 115), of whom 99% (n = 114) had a liver biopsy**.** Small duct PSC was diagnosed in a minority of patients (7%, n = 9), all of whom had a liver biopsy. The prevalence of IBD was 93% (n = 115); colectomy was performed in 10% (n = 12), of whom three had their colectomy before 18 years of age. Calprotectin was collected at IBD diagnosis and available in 41% (n = 47). Elevated calprotectin (range 166–9,421 mg/kg [normal <50 mg/kg]) was seen in all patients at diagnosis. Seventy percent (n = 86) had a low or medium SCOPE index at diagnosis. Detailed demographic and clinical characteristics at diagnosis are presented in Table 1.

Extrahepatic autoimmune comorbidities, other than IBD, were present in 21% of patients (n = 26: celiac disease [n = 9], thyroid disease [n = 6], psoriasis [n = 3], idiopathic thrombocytopenic purpura and antiphospholipid syndrome [both n = 2], with n = 1 for each of hemolytic anemia, IBD-associated arthritis, pyoderma gangrenosum, systemic lupus erythematosus, vasculitis, vitiligo and alopecia areata). Eight patients had more than one extrahepatic autoimmune comorbidity.

Treatment at last follow-up

At last follow-up, 56% (n = 69) were prescribed ursodeoxycholic acid (UDCA), 56% (n = 70) 5-ASA, 24% (n = 30) azathioprine, 4% (n = 5) vancomycin and 20% (n = 25) were treated with biologics (adalimumab [n = 8], infliximab [n = 6], vedolizumab [n = 6], ustekinumab [n = 5]). Azathioprine with or without additional prednisolone was used for the treatment of children with PSC and an additional diagnosis of AIH. UDCA was used in both subgroups (PSC 55% [n = 47], PSC and AIH 56% [n = 22], *p* = 1.000). There was a significant difference in treatment between the two subgroups regarding steroids (PSC 10% [n = 8], PSC and AIH 41% [n = 16], *p* <0.001) and azathioprine (PSC 18% [n = 15], PSC and AIH 38% [n = 15], *p* = 0.023).

Overall event-free survival

Overall event-free survival (from any of the outcomes) was 91% (95% CI 0.84–0.95) at 5 years and 77% (95% CI 0.68–0.84) at 10 years after diagnosis (Fig. 2A). Seventy-one percent of the patients did not meet any of the clinical outcomes during the follow-up time, median 13 years (5.7–21.6) (Table 2). No significant differences between the subgroups (PSC alone *vs.* PSC-AIH) were found in the univariable Cox proportional hazard model (Table 3). Time free from liver transplantation, biliary complications and portal hypertension are presented in Fig. 2B-D.

Portal hypertension

Five patients met the definition of portal hypertension at PSC diagnosis. Portal hypertension developed in 11% (n = 9) and 19% (n = 7) of the PSC alone and PSC-AIH groups during follow-up, respectively (Table 2). Ascites was present in 5% (n = 6), encephalopathy in 1% (n = 1), varices in 15% (n = 19) and splenomegaly with, at the same time, presence of low platelets in 9% (n = 11). Sixty-seven percent (n = 10) met more than one complication. Survival without portal hypertension was 95% (95% CI 0.89–0.98) 5 years and 90% (95% CI 0.82–0.94) 10 years after diagnosis (Fig. 2D). The Cox regression models showed no significant differences in outcome between the subgroups.

Biliary complications and ERCP

Biliary complications developed in 21% (n = 18) and 31% (n = 12) of patients with PSC or PSC-AIH during follow-up, respectively. Survival without biliary complications was 94% (95% CI 0.88–0.97) 5 years and 82% (95% CI 0.73–0.88) 10 years after diagnosis (Fig. 2C). Univariate and multivariate Cox proportional hazard model showed a decreased risk of developing biliary complications for girls (crude hazard ratio [HR] 0.40, 95% CI 0.17–0.94, *p* = 0.035), (adjusted HR 0.26, 95% CI 0.10–0.69, *p* = 0.007), and for ulcerative colitis *vs*. no IBD (crude HR 0.27, 95% CI 0.08–0.85, *p* = 0.026), (adjusted HR 0.16, 95% CI 0.04–0.60, *p* = 0.006) (Table 3). Thirty-two percent (n = 37) of the patients underwent at least one ERCP.

Hepatobiliary malignancies

No patient developed hepatobiliary malignancy during childhood, but two patients developed hepatobiliary malignancies later in life. One patient was diagnosed with CCA at the age of 28, fifteen years after PSC diagnosis and one with hepatocellular carcinoma (HCC) at the age of 23, twelve years after PSC diagnosis. Both patients had concomitant AIH and underwent liver transplantation and were alive 5 and 0.5 years, respectively, after cancer diagnosis.

Table 1. Demographic and clinical characteristics of the study cohort at diagnosis.

	Total N = 124	PSC n = 85	PSC and AIH n = 39
Sex male, % (n)	60 (75)	66 (56)	49 (19)
Age at diagnosis, year median (range)	14 (1.9–17.8)	13.8 (1.9–17.8)	14.4 (6.2–17.8)
Presence of IBD, % (n)	93 (115)	94 (80)	90 (35)
IBD phenotype, % (n)			
UC	54 (62/114)	54 (43/80)	56 (19/34)
CD	38 (43/114)	36 (29/80)	41 (14/34)
IBD-U	8 (9/114)	10 (8/80)	3 (1/34)
PSC phenotype, % (n)			
Large duct	93 (115/124)	92 (78/85)	95 (37/39)
Splenomegaly, % (n)	27 (27/99)	26 (17/66)	30 (10/33)
Stage of fibrosis, % (n)			
Mild (stage 0-2)	57 (66/115)	63 (52/82)	42 (14/33)
Severe (stage 3-4)	43 (49/115)	37 (30/82)	58 (19/33)
Grade of inflammation, % (n)			
0–1	72 (85/118)	85 (70/82)	42 (15/36)
2–4	28 (34/118)	16 (13/82)	58 (21/36)
Interface hepatitis, % (n)	23 (28/122)	14 (12/84)	42 (16/38)
Histological PSC changes, % (n)			
Typical	33 (40/123)	32 (27/84)	33 (13/39)
In line with	51 (63/123)	52 (44/84)	49 (19/39)
No changes	16 (20/123)	16 (13/84)	18 (7/39)
Total bilirubin median (range), mg/dl	0.41 (0.12–17)	0.41 (0.12–17)	0.44 (0.18–5.7)
GGT median (range), U/L	128 (7–1,037)	102 (7–894)	170 (8–1,037)
SCOPE index, % (n)			
Low (0-3 p)	38 (47)	42 (36)	28 (11)
Medium (4-5p)	32 (39)	26 (22)	44 (17)
High (6-11p)	14 (18)	14 (12)	15 (6)
Not assessable	16 (20)	18 (15)	13 (5)
Autoantibodies, % (n)			
Any	73 (62/85)	59 (29/49)	92 (33/36)
ANA	38 (41/109)	31 (22/71)	50 (19/38)
SMA	29 (34/117)	13 (10/78)	62 (24/399)
LKM	1 (1/89)	2 (1/60)	0 (0/29)
SLA	2 (1/63)	0 (0/40)	5 (1/22)
Any extrahepatic* autoimmune comorbidity, % (n)	21 (26/123)	23 (19/84)	18 (7/39)

AIH, autoimmune hepatitis; ANA, antinuclear antibody; CD, Crohn's disease; IBD, inflammatory bowel disease; IBD-U, IBD unclassified; LKM, liver/kidney microsome antibody; PSC, primary sclerosing cholangitis; SCOPE, Sclerosing Cholangitis Outcomes in Pediatrics; SLA, soluble liver antigen antibody; SMA, smooth muscle antibody; UC, ulcerative colitis.
*Other than IBD.

Liver transplantation, death and validation of SCOPE risk index

Thirteen percent of patients (n = 16) underwent liver transplantation, three before 18 years of age. Median time from diagnosis to liver transplantation was 7 (2.0–15.5) years. Transplant-free survival in the cohort was 97% (95% CI 0.92–0.99) at 5 years and 91% (95% CI 0.84–0.95) at 10 years after diagnosis (Fig. 2B). The univariate Cox regression model showed a tendency towards a higher risk of liver transplantation in the group with concomitant AIH (crude HR 2.85, 95% CI 1.06–7.67, p = 0.038) (Table 3, Fig. 3A). Three patients died during follow-up; only one death was liver related, occurring after transplantation.

SCOPE risk index (low/medium/high) at diagnosis predicted the risk of any hepatobiliary complication 5 years from diagnosis, with a c-statistic of 0.751 (HR 3.13, 95% CI 1.38–7.33), and liver transplantation or death at last follow-up, with a c-statistic of 0.70 (HR 2.350, 95% CI 1.18-4.66) (Table S1).

The proportion of patients who underwent liver transplantation was 4% (n = 2) in cases with low SCOPE index at diagnosis, 18% (n = 7) for median SCOPE index and 28% (n = 5) for high SCOPE index (Figs. 3B and 4).

Discussion

This retrospective study shows a very good long-term outcome in Swedish children diagnosed with PSC, with a transplant-free survival of 91% 10 years after diagnosis. No significant associations between sex, IBD status, age at diagnosis, and survival were found. The SCOPE index performed with reasonable accuracy (c-statistics = 0.70) for prediction of liver transplantation or death.

Few large longitudinal studies of pediatric PSC have been published.[3] We present one of the longest follow-ups (median 13 [5.7–21.6] years) published. To our knowledge this is also the largest single-center study published. Our data is in line with previous published papers with similar age at diagnosis, sex distribution, IBD and presence of AIH.[3,4,9–11] Earlier pediatric PSC studies have reported a prevalence of IBD of 76–90%.[3,4,9–11] Our cohort has a higher prevalence of Crohn's disease (CD) (38%) compared to previous papers (8–26%).[3,4,8–11] IBD in adult PSC is both clinically and genetically described as a distinct IBD phenotype when compared with IBD alone, with right sided colitis and a patchy inflammatory distribution. The PSC-IBD phenotype is probably common in children as well, which may be one reason for the high proportion of CD found here.[15] Categorizing PSC-colitis

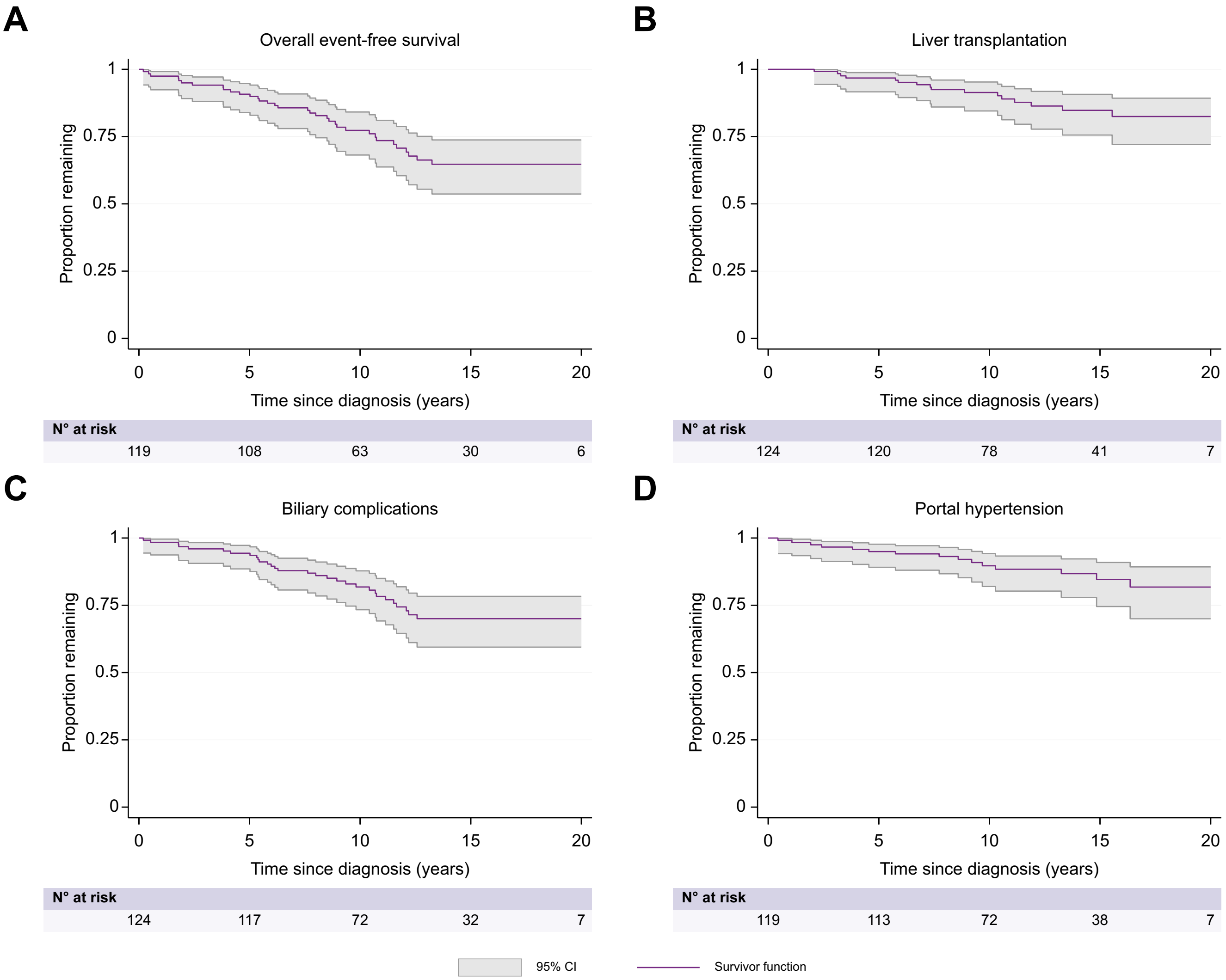

Fig. 2. Kaplan-Meier survival plots on the total cohort. (A) Overall event-free survival after diagnosis of PSC, defined as patients not meeting any of the outcomes of portal hypertension, biliary complications, hepatobiliary cancer, liver transplantation or death by liver disease. (B) Liver transplantation after diagnosis of PSC. (C) Development of biliary complications in need of intervention after diagnosis of PSC. (D) Development of portal hypertension, defined as at least one of the following: ascites in need of medical treatment or intervention, hepatic encephalopathy, esophageal varices or splenomegaly and at the same time low platelet count after diagnosis of PSC. PSC, primary sclerosing cholangitis.

as ulcerative colitis or CD may therefore be arbitrary and therefore should be avoided.

We used the simplified diagnostic criteria for AIH validated for children to establish an additional diagnosis of AIH in our patients with PSC.[14] AIH was present in 31%, comparable to previous reports using the same criteria.[3,4,9,10] AIH type 2 is considerably more uncommon in children compared to AIH type 1.[16] No one in our cohort had a diagnosis of AIH type 2. This might be explained by the high presence of IBD, which is mainly associated with AIH type 1. There is an ongoing

Table 2. Clinical long-term outcomes in the cohort and in the two subgroups separately.

Outcomes	Total N = 124	PSC n = 85	PSC and AIH n = 39	Fisher's exact test *p* value
Portal hypertension, % (n/N)	13 (16/119)	11 (9/82)	19 (7/37)	0.256
Biliary complication, % (n/N)	24 (30/124)	21 (18/85)	31 (12/39)	0.265
HB cancer, % (n/N)	2 (2/124)	0 (0/85)	5 (2/39)	0.097
Liver transplantation, % (n/N)	13 (16/124)	8 (7/85)	23 (9/39)	**0.040**
Death due to liver disease, % (n/N)	1 (1/124)	1 (1/85)	0 (0/39)	1.000
Overall event-free survival, % (n/N)	71 (84/119)	74 (61/82)	62 (23/37)	0.197

AIH, autoimmune hepatitis; HB, hepatobiliary; PSC, primary sclerosing cholangitis.
Outcomes between the subgroups were compared with Fisher's exact test and *p* value <0.05 was considered significant. Significant *p* values are in bold. Median time of follow-up 13 (5.7–21.6) years. N = number of observations.

Table 3. Univariate and multivariate Cox proportional hazards model for overall event-free survival, liver transplantation, and biliary complications.

			Univariable			Multivariable		
**Characteristic**	**N**	**Event N**	**Crude HR**	**95% CI**	***p* value**	**adj HR**	**95% CI**	***p* value**
**Overall event-free survival**								
Sex	119	35			0.085			0.034
Male			–	–	–	–	–	–
Female			0.54	0.26-1.12	0.10	0.43	0.19–0.97	**0.042**
Age at diagnosis	119	35						
≤12 years			–	–	–	–	–	–
>12 years			1.57	0.73–3.36	0.2	1.22	0.56–2.68	0.6
PSC phenotype	117	35			0.4			0.3
Large duct			–	–	–	–	–	–
Small duct			0.46	0.06–3.34	0.4	0.40	0.05–2.99	0.4
Concomitant AIH	119	35			0.3			0.2
No			–	–	–	–	–	–
Yes			1.42	0.72–2.80	0.3	1.55	0.74–3.24	0.2
IBD phenotype	119	35			0.6			0.6
No IBD			–	–	–	–	–	–
Ulcerative colitis			0.57	0.16–1.97	0.4	0.47	0.13–1.78	0.3
Crohn's disease			0.89	0.26–3.12	0.9	0.68	0.17–2.67	0.6
IBD-U			0.96	0.19–4.78	>0.9	0.80	0.15–4.37	0.8
GGT	115	35						
≤36.0 U/L			–	–	–	–	–	–
>36.0 U/L			1.27	0.63–2.54	0.5	1.16	0.55–2.44	0.7
**Liver transplantation**								
Sex	124	16			0.8			0.3
Male			–	–	–	–	–	–
Female			0.86	0.31–2.36	0.8	0.57	0.18–1.83	0.3
Age at diagnosis	124	16						
≤12 years			–	–	–	–	–	–
>12 years			1.21	0.42–3.49	0.7	1.02	0.34–3.03	> 0.9
PSC phenotype	122	16			0.2			0.14
Large duct			–	–	–	–	–	–
Small duct			0.00	0.00–Inf	>0.9	0.00	0.00–Inf	>0.9
Concomitant AIH	124	16			**0.038**			0.2
No			–	–	–	–	–	–
Yes			2.85	1.06–7.67	**0.038**	1.99	0.71–5.63	0.2
IBD phenotype	124	16			0.13			0.14
No IBD			–	–	–	–	–	–
Ulcerative colitis			0.26	0.07–1.02	0.053	0.25	0.06–1.13	0.072
Crohn's disease			0.36	0.09–1.44	1.44	0.36	0.07–1.76	0.2
IBD-U			0.00	0.00–Inf	>0.9	0.00	0.00–Inf	>0.9
GGT	120	16						
≤36.0 U/L			–	–		–	–	–
>36.0 U/L			2.40	0.78–7.46	0.11	2.33	0.71–7.73	0.15
**Biliary complications**								
Sex	124	30			**0.024**			**0.003**
Male			–	–	–	–	–	–
Female			0.40	0.17–0.94	**0.035**	0.26	0.10–0.69	**0.007**
Age at diagnosis	124	30						
≤12 years			–	–	–	–	–	–
>12 years			1.48	0.66–3.33	0.3	0.98	0.42–2.27	>0.9
PSC phenotype	122	30			0.6			0.3
Large duct			–	–	–	–	–	–
Small duct			0.59	0.08–4.35	0.6	0.38	0.05–3.03	0.4
Concomitant AIH	124	30			0.3			0.2
No			–	–	–	–	–	–
Yes			1.47	0.71–3.05	0.3	1.62	0.73–3.58	0.2
IBD phenotype	124	30			0.083			**0.043**
No IBD			–	–	–	–	–	–
Ulcerative colitis			0.27	0.08–0.85	**0.026**	0.16	0.04–0.60	**0.006**
Crohn's disease			0.60	0.20–1.86	0.4	0.32	0.09–1.23	0.10
IBD-U			0.66	0.15–2.96	0.6	0.45	0.09–2.35	0.3
GGT	120	30						
≤36.0 U/L			–	–		–	–	–
>36.0 U/L			1.43	0.68–3.00	0.3	1.28	0.57–2.88	0.5

AIH, autoimmune hepatitis; GGT, gamma-glutamyltransferase; HR, hazard ratio; IBD, inflammatory bowel disease; IBD-U, IBD unclassified; PSC, primary sclerosing cholangitis. *p* values <0.05 were considered significant. Significant *p* values are in bold.

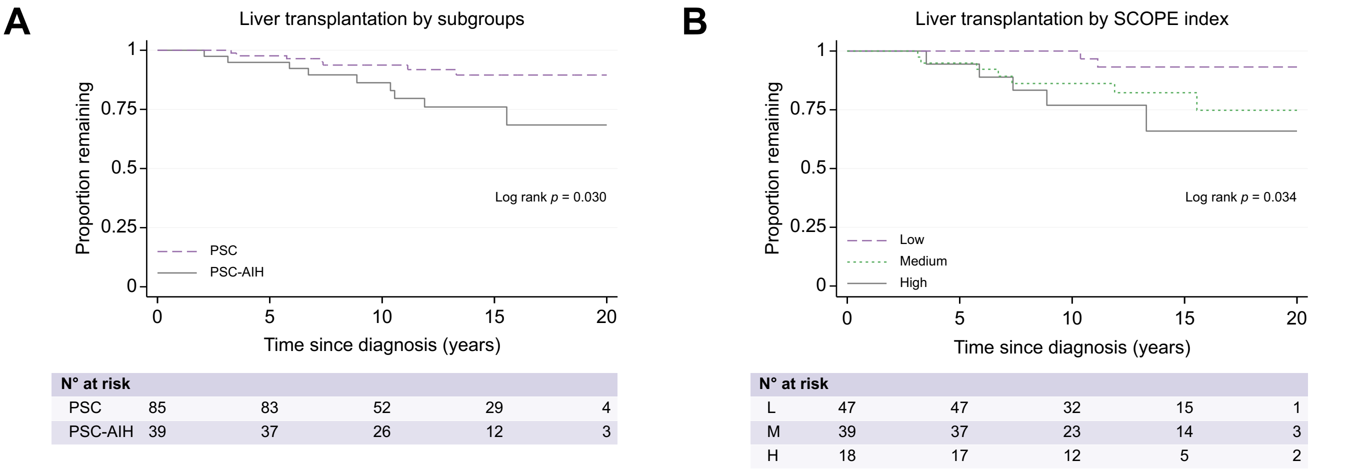

Fig. 3. Survival plots on liver transplantation during follow-up. (A) Kaplan-Meier curves and log-rank test for performed liver transplantations divided by the two subgroups (PSC *vs.* PSC-AIH). AIH, autoimmune hepatitis; PSC, primary sclerosing cholangitis; SCOPE, Sclerosing Cholangitis Outcomes in Pediatrics. (B) Kaplan-Meier curves and log-rank test for liver transplantation divided by low/medium/high SCOPE risk index at diagnosis.

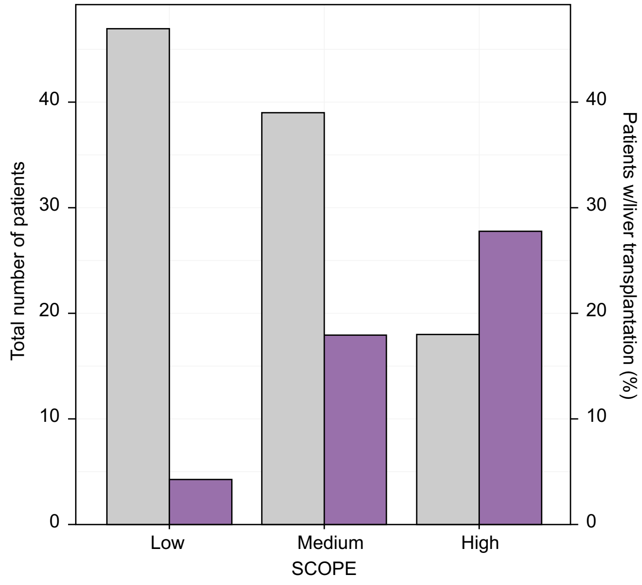

Fig. 4. Frequency of liver transplantations at last date of follow-up divided by SCOPE index at diagnosis. Median time of follow-up 13 (5.7–21.6) years. Gray bars = total number of patients in each group; Purple bars = % of liver transplantations in each group; Low risk = 0-3 points, medium risk = 4-5 points, high risk = 6-11 points (Cox proportional hazard regression, hazard ratio = 2.350, 95% CI 1.18–4.66, *p* value = 0.015, c-statistic = 0.701). SCOPE, Sclerosing Cholangitis Outcomes in Pediatrics.

discussion on whether PSC with AIH in children represents an early inflammatory phase of PSC.[17,18] We found a slightly increased need for liver transplantation in patients with PSC-AIH compared to PSC alone, which has not previously been reported.[3,4,10] However, this data should be interpreted with caution due to the low number of events. The reason for this finding may be influenced by the high fibrosis stage at inclusion in this subpopulation.

Previous pediatric studies of PSC have reported a poorer outcome than shown in the present study, despite shorter follow-up times. Deneau *et al.* reported liver transplantation in 14% of cases during a median time of follow-up of 4.4 years.[3] The clinical characteristics at diagnosis between the multicenter cohort and the cohort in this study are similar regarding presence of portal hypertension and AIH, whereas our cohort has a higher presence of IBD (93% *vs.* 76%) but a lower presence of small duct phenotype (7% *vs.* 13%). Considering the clinical characteristics, one might have expected a poorer outcome in our cohort. Valentino *et al.* reported liver transplantation in 5% over a median follow-up of 3.7 years.[10] A recently published multicenter study reported liver transplantation in 6% at 3 years after diagnosis in a cohort of pediatric patients with PSC-IBD.[19] No significant difference in liver transplantation was seen depending on age (<6 *vs.* 6–18 years of age) at PSC diagnosis. Possible explanations for the better outcome in our cohort can only be speculated upon. Our children may have been diagnosed at an earlier stage compared with other studies. However, our center is a tertiary referral center, and significant fibrosis (stage 3–4) was found in 43% at diagnosis.

PSC-related cholangiocarcinoma is rarely seen in children, and none in this cohort were diagnosed with CCA during childhood. Only one patient was diagnosed with CCA during follow-up, which is lower than in previous reports.[3,4]

It has been proposed that the SCOPE index be used as a complement to clinical evaluation in children with PSC.[12,20] In a recent study, the SCOPE index was shown to be more likely to better predict development of complications when calculated 5 years after diagnosis than at diagnosis.[8] We tested the performance of the SCOPE index at 5 years and at last follow-up and we found a reasonable accuracy regarding liver transplantation or death at last follow-up (c-statistic 0.70). However, this result must be interpreted with caution due to the very low number of events. Its role in clinical practice in individual prognostication is yet to be determined.

Earlier studies have shown normalization of liver enzymes with UDCA at doses of 13–15 mg/kg/day, but no effect on long-term outcome.[21] A recent retrospective study, matched for baseline characteristics, has shown no difference in outcome between treatment with UDCA compared to observation (no

treatment at all) or treatment with oral vancomycin.[22] Liver enzymes in PSC fluctuate naturally during disease-course and spontaneous normalization is not unusual.[22] In our cohort, 48% (n = 60) of patients continued treatment with UDCA after 18 years of age and 4% (n = 5) of patients were treated with oral vancomycin. We were, however, not able to assess the impact of UDCA or vancomycin in this retrospective setting.

A strength of this study is the relatively large number of patients and long follow-up time, in comparison to previous studies.[3,4,8,10,11] Only one study has reported a follow-up approaching the time presented in our study, with a median time of follow-up of 12 (4–17) years in a cohort of 25 patients.[9]

This being a retrospective study, it has obvious limitations including missing variables. Despite a relatively large number of patients, the study has too few events to draw clear conclusions. Moreover, medical treatment could not be completely assessed, and changes in treatment of IBD or other autoimmune diseases over time may have influenced the outcomes.

In this first longitudinal study of Swedish patients with PSC diagnosed in childhood, we found an unexpectedly good prognosis in young individuals who were followed into adulthood in most cases. There is a great need among children with PSC and their parents for more knowledge about the natural history of this disease and what they can expect from the future. We hope that the data presented in this study may help counsel health professionals, young individuals and families affected by this disease.

Affiliations

[1]*Department of Medicine Huddinge, Karolinska Institutet, Department of Acute Geriatrics, Stroke and Palliative care, Nyköping Hospital Nyköping, Sweden;* [2]*Department of Clinical Science, Intervention and Technology (CLINTEC), Unit of Pediatrics, Karolinska Institutet, Pediatric Gastroenterology, Hepatology and Nutrition, Karolinska University Hospital, Stockholm, Sweden;* [3]*Department of Medicine Huddinge, Karolinska Institutet, Department of Upper GI Disease, Division of Hepatology, Karolinska University Hospital, Stockholm, Sweden;* [4]*Department of Medicine Huddinge, Karolinska Institutet, Department of Gastroenterology, Dermatovenereology and Rheumatology, Centre for Digestive Health, Karolinska University Hospital, Stockholm, Sweden*

Abbreviations

AIH, autoimmune hepatitis; CCA, cholangiocarcinoma; CD, Crohńs disease; ERCP, endoscopic retrograde cholangiopancreatography; GGT, gamma-glutamyltransferase; HCC, hepatocellular carcinoma; IBD, inflammatory bowel disease; MRCP, magnetic resonance cholangiopancreatography; PSC, primary sclerosing cholangitis; SCOPE, Sclerosing Cholangitis Outcomes in Pediatrics; UC, ulcerative colitis; UDCA, ursodeoxycholic acid.

Financial support

The study has received funding from Centre for Clinical Research Sörmland, Uppsala University, Sweden and The Sven Jerring Foundation, Stockholm, Sweden.

Conflict of interest

No: Lina Lindström, Annika Bergquist, Thomas Casswall, Anna Jerregård Skarby. Please refer to the accompanying ICMJE disclosure forms for further details.

Authors' contributions

Study design and concept AB, TC, LL, AJS, analysis and interpretation of results AJS, AB, LL and TC, first draft manuscript preparation AJS, all authors reviewed the results and approved the final version of the manuscript.

Data availability statement

Data are available at reasonable request to the corresponding author.

Acknowledgements

Statisticians Laura Vossen Engblom and Raphaela Mayerhofer kindly provided statistical advice for the manuscript.

Supplementary data

Supplementary data to this article can be found online at https://doi.org/10.1016/j.jhepr.2024.101123.

References

Author names in bold designate shared co-first authorship

[1] Karlsen TH, Folseraas T, Thorburn D, et al. Primary sclerosing cholangitis - a comprehensive review. J Hepatol 2017;67(6):1298–1323.
[2] Boonstra K, Weersma RK, van Erpecum KJ, et al. Population-based epidemiology, malignancy risk, and outcome of primary sclerosing cholangitis. Hepatology 2013;58(6):2045–2055.
[3] Deneau MR, El-Matary W, Valentino PL, et al. The natural history of primary sclerosing cholangitis in 781 children: a multicenter, international collaboration. Hepatology 2017;66(2):518–527.
[4] Deneau M, Jensen MK, Holmen J, et al. Primary sclerosing cholangitis, autoimmune hepatitis, and overlap in Utah children: epidemiology and natural history. Hepatology 2013;58(4):1392–1400.
[5] Boonstra K, van Erpecum KJ, van Nieuwkerk KM, et al. Primary sclerosing cholangitis is associated with a distinct phenotype of inflammatory bowel disease. Inflamm Bowel Dis 2012;18(12):2270–2276.
[6] **Weismüller TJ**, **Trivedi PJ**, Bergquist A, et al. Patient age, sex, and inflammatory bowel disease phenotype associate with course of primary sclerosing cholangitis. Gastroenterology 2017;152(8):1975. 84.e8.
[7] Adike A, Carey EJ, Lindor KD. Primary sclerosing cholangitis in children versus adults: lessons for the clinic. Expert Rev Gastroenterol Hepatol 2018;12(10):1025–1032.
[8] Hensel KO, Kyrana E, Hadzic N, et al. Sclerosing cholangitis in pediatric inflammatory bowel disease: early diagnosis and management affect clinical outcome. J Pediatr 2021;238:50. 6.e3.
[9] Smolka V, Karaskova E, Tkachyk O, et al. Long-term follow-up of children and adolescents with primary sclerosing cholangitis and autoimmune sclerosing cholangitis. Hepatobiliary Pancreat Dis Int 2016;15(4):412–418.
[10] Valentino PL, Wiggins S, Harney S, et al. The natural history of primary sclerosing cholangitis in children: a large single-center longitudinal cohort study. J Pediatr Gastroenterol Nutr 2016;63(6):603–609.
[11] Tenca A, Färkkilä M, Arola J, et al. Clinical course and prognosis of pediatric-onset primary sclerosing cholangitis. United Eur Gastroenterol J 2016;4(4):562–569.
[12] Deneau MR, Mack C, Perito ER, et al. The sclerosing cholangitis outcomes in pediatrics (SCOPE) index: a prognostic tool for children. Hepatology 2021;73(3):1074–1087.
[13] EASL clinical practice guidelines on sclerosing cholangitis. J Hepatol 2022;77(3):761–806.
[14] Mileti E, Rosenthal P, Peters MG. Validation and modification of simplified diagnostic criteria for autoimmune hepatitis in children. Clin Gastroenterol Hepatol 2012;10(4):417. 421 e1-2.
[15] Ricciuto A, Kamath BM, Griffiths AM. The IBD and PSC phenotypes of PSC-IBD. Curr Gastroenterol Rep 2018;20(4):16.
[16] Jiménez-Rivera C, Ling SC, Ahmed N, et al. Incidence and characteristics of autoimmune hepatitis. Pediatrics 2015; 2015;136(5):e1237. 48.
[17] Ricciuto A, Kamath BM, Hirschfield GM, et al. Primary sclerosing cholangitis and overlap features of autoimmune hepatitis. A Coming Age Or Age-ist Problem? J Hepatol 2023;79(2):567. 75.
[18] Mieli-Vergani G, Vergani D, Baumann U, et al. Diagnosis and management of pediatric autoimmune liver disease: ESPGHAN hepatology committee position statement. J Pediatr Gastroenterol Nutr 2018;66(2):345–360.
[19] Catassi G, D'Arcangelo G, Norsa L, et al. Outcome of very early onset inflammatory bowel disease associated with primary sclerosing cholangitis: a

multicenter study from the pediatric IBD porto Group of ESPGHAN. Inflamm Bowel Dis 2023 Sep 28:izad218.
[20] Fuchs Y, Valentino PL. Natural history and prognosis of pediatric PSC with updates on management. Clin Liver Dis (Hoboken) 2023;21(2):47–51.
[21] Floreani A, De Martin S. Treatment of primary sclerosing cholangitis. Dig Liver Dis 2021;53(12):1531–1538.
[22] Deneau MR, Mack C, Mogul D, et al. Oral vancomycin, ursodeoxycholic acid, or No therapy for pediatric primary sclerosing cholangitis: a matched analysis. Hepatology 2021;73(3):1061–1073.

Keywords: PSC; Pediatric liver disease; SCOPE index.
Received 17 November 2023; received in revised form 14 May 2024; accepted 21 May 2024; Available online 25 May 2024

Journal of Hepatology, Volume *6*

Supplemental information

Good long-term outcomes of primary sclerosing cholangitis in childhood

Anna Jerregård Skarby, Thomas Casswall, Annika Bergquist, and Lina Lindström

Good long-term outcomes of primary sclerosing cholangitis in childhood

Anna Jerregård Skarby, Thomas Casswall, Annika Bergquist, Lina Lindström

Table of contents

Table S1……………………………………………………………………………...….2

Table S1. Cox PH regression with c-statistics to evaluate performance of SCOPE risk index at 5 years from diagnosis of PSC and at last date of follow-up.

	5 years after diagnosis				Last follow-up			
	**HR**	**95% CI**	***p*-value**	**c-statistics**	**HR**	**95% CI**	***p*-value**	**c-statistics**
**Any hepatobiliary complications**	3.131	1.38-7.33	**0.009**	0.751	1.380	0.88 - 2.19	0.171	0.590
**Liver transplantation/ Death from liver disease**	2.873	0.61-13.59	0.183	0.732	2.350	1.18 - 4.66	**0.015**	0.701

SCOPE index = Sclerosing Cholangitis Outcomes in Pediatrics index, Any hepatobiliary complications = any of portal hypertension, biliary complications, hepatobiliary cancer, liver transplantation or death of liver disease, HR = Hazard ratio, CI = confidence interval, c-statistic = concordant statistic
p value <0.05 was considered significant. Significant *p* values are in bold.
